# MxArray: A Modular, Multiplexed, and Massive MEMS-Based Acoustic Array

**DOI:** 10.3390/s26123899

**Published:** 2026-06-19

**Authors:** Ricardo Moreno, Jorge Ortigoso-Narro, Daniel de la Prida, Luis A. Azpicueta-Ruiz, Borja Genovés Guzmán, Marco Raiola

**Affiliations:** 1 Department of Aerospace Engineering, Universidad Carlos III de Madrid, 28911 Leganés, Spain; mraiola@ing.uc3m.es; 2 Department of Electronic Technology, Universidad Carlos III de Madrid, 28911 Leganés, Spain; jortigos@pa.uc3m.es; 3 Grupo de Investigación en Acústica Arquitectónica, Universidad Politécnica de Madrid, 28040 Madrid, Spain; daniel.prida@upm.es; 4 Department of Signal Theory and Communications, Universidad Carlos III de Madrid, 28911 Leganés, Spain; lazpicue@ing.uc3m.es (L.A.A.-R.); bgenoves@ing.uc3m.es (B.G.G.)

**Keywords:** embedded system, massive acoustic array, MEMS microphone array, multichannel audio serial port, time-division multiplexing

## Abstract

**Highlights:**

**What are the main findings?**

- The research and development of a scalable 1024-MEMS microphone array using a distributed network of 16 BeagleBone Black modules and TDM architecture.
- Microsecond synchronization across the distributed nodes is achieved using PTP and PHC discipline with a priority-scheduled pulse injection strategy.

**What are the implications of the main findings?**

- Low-cost embedded Linux systems with PREEMPT_RT kernels are a viable alternative for massive acoustic sensing.
- The cost and complexity of high-resolution aeroacoustics imaging are reduced, making massive arrays accessible for standard research facilities.

**Abstract:**

While state-of-the-art massive acoustic arrays typically rely on costly, specialized FPGA architectures or rigid proprietary hardware, there is a growing need for modular, high-density sensing in complex aeroacoustics environments. This paper presents the electronic and acoustic design of a multiplexed, modular, scalable, and low-cost massive acoustic array (MxArray) founded on an embedded Linux system. The AM3358 SoC microprocessor collects audio data through its multichannel audio peripheral, where it simultaneously receives four Time-Division Multiplexing streams of 16 microphones each. This multiplexed scheme enables the handling of 64 microphones per module, whose acquisition synchronization is set with the Precision Time Protocol and a pulse injection hardware. The combination of both BeagleBone Black and microphones based on Micro-Electro-Mechanical Systems yields a cost-effective solution with built-in Ethernet connectivity and accessible software development through an embedded Linux environment with audio libraries for hardware control. Sensors are arranged in an Underbrink Spiral pattern on a four-layer printed-circuit board. The perforated thin layout minimizes any airborne disturbance, exploiting a distribution that simultaneously achieves a low sidelobe level and a narrow main lobe when used with a beamforming algorithm. Measurement results for the developed module are presented, as well as an evaluation of a full-scale system comprising 16 modules (1024 microphones) arranged in a honeycomb pattern. The resulting instrument offers a practical and scalable solution for applications that require a large number of simultaneous microphone measurements, such as beamforming technology for aeroacoustics applications.

## 1. Introduction

Phased microphone arrays are widely used as diagnostic tools in engineering and scientific fields where identifying the location of sound sources is essential. Since 1974, these systems, combined with beamforming algorithms, have enabled the localization and tracking of moving sound sources even in challenging environments [1]. By processing multiple audio signals acquired simultaneously, it is possible to estimate the positions of one or more sound sources.

Although the need for large arrays is well-established [2], conventional acoustic systems installed in research facilities face three critical drawbacks: affordability, scalability, and accessibility. In the past, traditional acquisition systems comprised an array of condenser microphones, a multichannel preamplifier, and a custom-built multichannel analog-to-digital converter (ADC) [3], where the cost of these systems increased linearly with the channel count. Additionally, to obtain a high-resolution multichannel recording, centralized hardware synchronization with rigid clock distribution networks was needed to achieve the phase accuracy required for coherent beamforming. This results in high engineering costs and limited design flexibility, especially if the microphones are fixed in a structure, making them non-modular and difficult to deploy in diverse environments.

Conversely, the advent of Micro-Electro-Mechanical Systems (MEMS) technology has considerably changed the paradigm of massive acoustic sensing. Its advantages include miniature size, digital audio transmission, simplified electrical connection, and a substantial budget reduction. A large number of electronic sensors can be wired by soldering dense connectors over an Integrated Circuit (IC), which is often handled by a Field Programmable Gate Array (FPGA) for parallel data acquisition [4]. Despite their parallel processing capabilities, FPGA-based arrays introduce significant challenges affecting their implementation, affordability, and scalability. These systems demand specialized programming using hardware description languages, and significantly increase the development effort and the expertise required by a highly qualified research team. Furthermore, hardware implementation and scaling of FPGA arrays typically incur high costs due to the need for advanced chips. Besides, they often necessitate expensive commercial integration platforms [5]. These factors create an accessibility gap, pushing up both the capital costs and engineering labor of building large-channel-count systems.

Recent MEMS microphone array designs increasingly use serial digital audio interfaces such as the Inter-IC Sound ($I^{2}S$) protocol [6]. By allowing a pair of microphones to share a single data line, this interface enables compact microcontrollers, such as the Arduino Nano [7], to acquire stereo audio efficiently. However, the standard $I^{2}S$ protocol supports only two audio channels per data line, which limits its suitability for large-scale sensing applications by significantly increasing routing requirements and overall circuit complexity. To overcome these limitations, Time-Division Multiplexing (TDM) can be used to extend $I^{2}S$, allowing multiple independent audio channels to be transmitted over a single serial data line. By using low-power microphones with digital outputs, this approach reduces wiring density, simplifies circuit design, and enables scalable, high-channel-count microphone arrays.

Thus, scalability, electronic complexity, and cost-related factors have created a persistent gap in both traditional setups and MEMS-based ICs. As a result, high-resolution acoustic imagery is dominated by a small number of well-funded research groups utilizing specialized, high-cost instrumentation. In order to overcome these constraints and propose a new benchmark for scalable acoustic imaging, this study introduces the MxArray: a novel MultipleXed, Modular, Massive, LinuX-embedded, MEMS-based Acoustic Array. This architecture skips the centralized analog acquisition by combining the modularity of a synchronized network of development boards with the cost-effectiveness of MEMS sensors. The microphone’s TDM digital output stream simplifies the system by entrusting processes such as transduction, Analog-to-Digital conversion, and multiplexing to the MEMS’ Application-Specific Integrated Circuit (ASIC) chip. The core innovation of the MxArray is its modular scalability. Its architecture allows synchronization and data processing in modules of 64 channels up to the network capacity, using a highly distributed, embedded Linux system approach. This is achieved through three integrated strategies:

1. Scalable Synchronization: Utilizing open, low-cost Ethernet-based network protocols namely, Precision Time Protocol (PTP) and Message Queuing Telemetry Transport (MQTT)—for inter-module synchronization.
2. Efficient Data Packaging: Implementing TDM on compact, modular 64-channel acquisition boards.
3. Optimized Spatial Sampling: Integrating a non-redundant, log-spiral sensor distribution directly onto the Printed Circuit Board (PCB) modules.

The main objective of this research is to design, prototype, and validate the MxArray to demonstrate that a modular, low-cost platform can deliver phase coherence and beamforming performance comparable to state-of-the-art acquisition systems. By optimizing the embedded Linux resource management, the system achieves massive parallel acquisition that approaches the synchronization accuracy of FPGA-based architectures while significantly reducing programming complexity and hardware costs.

The remainder of this manuscript is organized as follows: Section 2 outlines the design process based on the management of multiplexed signals. In Section 3, the development board used for each module, along with its respective modifications, is described. The modular synchronization is achieved through the injection of an electric pulse, as explained in Section 4. Section 5 details the methodology employed to assess the system’s temporal response and synchronization precision. The experimental results and a comprehensive discussion of the findings are presented in Section 6 and Section 7, respectively. Finally, Section 8 provides our concluding remarks and directions for future work.

## 2. Concept of Design

**Figure 1:**
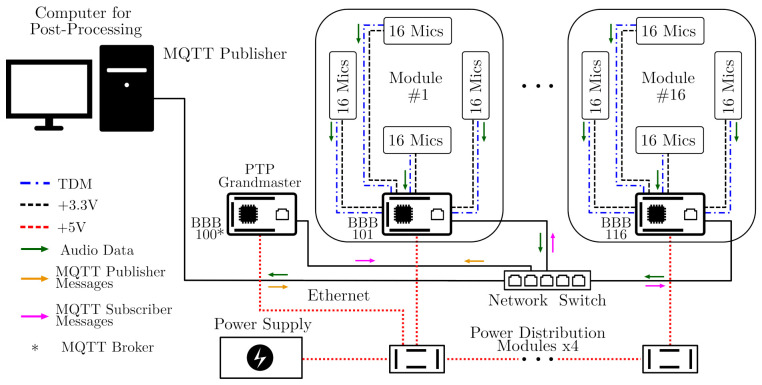
High-level diagram of the complete system architecture and the connections involved.

This section introduces the overall concept and design principles supporting the system. The complete high-level architecture of the MxArray, shown in Figure 1, illustrates the interaction between the different hardware and control blocks, as well as the data flow from the MEMS microphones to the computer.

Each module consists of four groups of 16 microphones, controlled by a development board. An additional board (BBB 100) is used to synchronize the system and to act as a message broker for receiving the command from the computer and sending it to the 16 boards. Once the recording has finished, the computer pulls the 64-channel file located in each BBB. This design emphasizes modularity, synchronization, and scalability to support multiple audio channels efficiently. It also ensures electrical stability across the system through power distribution.

### 2.1. TDM Protocol

**Figure 2:**
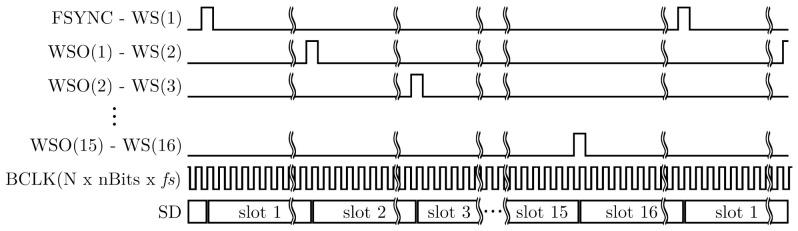
TDM Transmission diagram showing the FSYNC signal synchronization and propagation along the daisy-chain system until the 16th microphone. Data bits of each microphone are grouped into slots, commonly referred to as ”channels” in TDM terminology. One frame consists of 16 slots. Once the previous microphone finishes writing its slot, it sends the pulse through WSO terminal to the next WS microphone pin.

**Figure 3:**
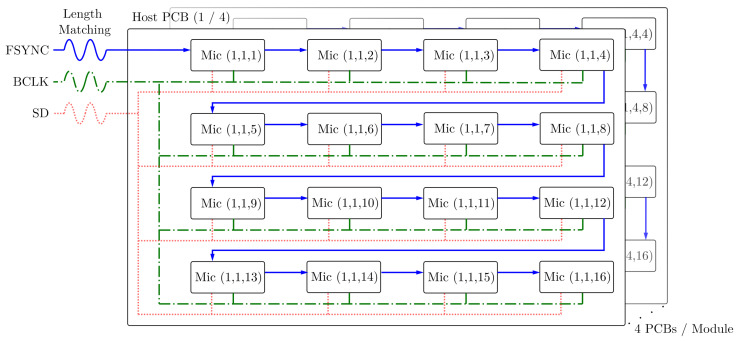
Connections of the digital lines of the data interface for a sixteen-microphone sub-module. Each microphone is indexed as (S,P,N), where *S* is the number of spiral modules, *P* is the number of the PCB inside the spiral, and *N* is the microphone inside each sub-module.

A key feature of the modular design is the use of multiplexed audio streams provided by TDM-enabled MEMS microphones, which optimizes the number of data lines and simplifies electronic interconnections on the integrated circuit. In addition to its low cost, the TDK ICS-52000 microphone (InvenSense Inc., San Jose, CA, USA) saves the need for an audio codec to convert the electric signal into PCM and manage channel multiplexing [8], exploiting the full potential of the array configuration [9]. The selected sensor, developed in 2016, was the leading option available on the market capable of streaming up to 16 audio signals through a single data line, eliminating the need for additional interfaces. Figure 2 depicts the signal flow of the microphone array cascade connection. Together with the supply voltage and ground lines, the TDM interface requires two more synchronized signals to activate the microphones and stream audio data sequentially: the bit clock (BCLK) and the frame sync (FSYNC). BCLK provides the timing at which data payload is written, and its oscillation frequency is the product of the number of microphones (*N*), the sampling rate ($f_{s}$), and the number of bits (nBits) per slot. For instance, to capture sound with 16 microphones at a sampling rate of 8 kHz and 32-bit word length, the TDM interface would require a bit clock of 4.096 MHz. The second synchronized signal, FSYNC, is the conductor that drives the first microphone in the daisy-chain at the same frequency of $f_{s}$. This short pulse is propagated across the rest of the microphones, connecting every microphone’s Word Select Output (WSO) to the Word Select (WS) input pin of the subsequent element, effectively acting as a relay that grants permission to place data onto the bus. In addition, the ICS-52000 Serial Data (SD) bus gathers PCM audio in Big-Endian order and two’s complement binary encoding formatted as a 32-bit word. In practice, each microphone outputs 24-bit audio data, and the eight subsequent bits are zero-padded. Figure 3 illustrates the connection schematic for the complete 16-microphone sub-module.

### 2.2. PCB Characteristics

**Figure 4:**
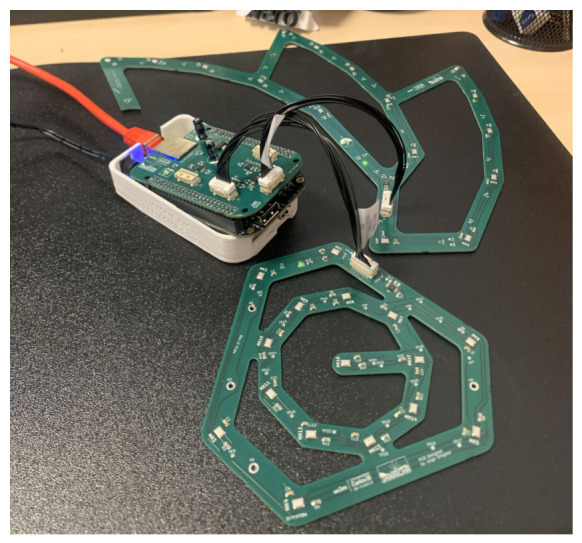
BeagleBone Black cape, spiral center, and spiral arms prototypes.

The PCB development process involved two main prototyping iterations. As a proof-of-concept, the first uniform planar array prototype successfully demonstrated that connecting 16 TDM MEMS microphones was feasible, confirming reliable multiplexing performance. Given that the TDM interface requires a BCLK on the order of megahertz, reflections and ringing in the SD signal must be avoided. The SD trace length was deliberately extended to the maximum recommended by the manufacturer to avoid both excessive propagation delay and data misalignment [10]. These initial experiments revealed minor inherent variations among microphones, particularly in their acoustic frequency response [11]. Furthermore, the four-layer PCB design incorporated a permeable structure to minimize air incidence, which helps reduce both aerodynamic interference and sound reflections that could otherwise affect the acoustic response of the array and create potential flow disturbances when deployed in testing environments. This is relevant to our case, which involves aeroacoustic noise sources in jet facilities [12]. The physical properties of the PCB must facilitate manufacturing efficiency and cost-effectiveness while ensuring practical part replacement and maintained signal integrity. To address these aspects, we implemented controlled trace dimensions and ground referencing to help with impedance matching, whilst termination resistors were added along the shared data bus to prevent ringing, overshooting, and reflections. As illustrated in Figure 4, the second prototype included connectors over the cape (see Section 2.5) and the spiral parts.

### 2.3. BeagleBone Black

Following a comprehensive survey of available development boards, the BeagleBone^®^ Black (BBB) (BeagleBoard.org Foundation, Oakland Township, MI, USA) [13] was selected as the platform that best satisfies the technical requirements while maintaining a low budget per module. In the BBB’s last revision (RevC3), an AM3358 Sitara microprocessor [14] was incorporated, whose audio peripheral makes it suitable for multichannel audio applications. The processor’s audio interface is managed by the Multichannel Audio Serial Port (McASP) unit, and it offers multiple serializers either for receiving or transmitting audio data. This dedicated peripheral unit is embedded in the AM3358 Texas Instruments^®^ System on Chip (SoC) (Texas Instruments, Dallas, TX, USA), and it interconnects McASP with an ARM Cortex-A8 CPU. Other designs have shown the feasibility of daisy-chaining of the 16 ICS-52000 units, with more robust platforms such as Jetson Orin Nano [15], but this board offers other hardware elements not related with the acquisition that incur a rise in the budget.

Other components on the BBB board are also used, including an external quartz oscillator, which is used to feed the McASP unit. In the default configuration, this oscillator allows the HDMI service to send audio signals at a frequency of 24.576 MHz, which leads to a convenient sample rate for our 16-microphone multiplexing with an integer division. This signal clock is set as a McASP external source and divided down to obtain both BCLK and FSYNC signals.

### 2.4. Sensor Distribution

The spatial distribution of the sensors is crucial for achieving accurate source localization. Issues such as grating lobes and spatial aliasing can be mitigated by ensuring that the spacing between microphones is non-redundant and as diverse as possible [16]. For this reason, the sensor layout for each module follows the Underbrink pattern, as it is known to be one of the most efficient concentric configurations [17]. This pattern minimizes spatial aliasing and spurious artifacts in the acoustic map by employing a geometry with circular symmetry. Sensors are positioned at varying arc lengths and radii along uniformly spaced logarithmic spirals centered around a common origin. An odd number of spiral arms is preferred to eliminate redundancy in microphone spacing, thereby reducing the risk of spatial aliasing [16]. Therefore, the radial and angular coordinates of the microphone located at the *n*-th ring and the *m*-th spiral arm are computed as

(1) $$r_{m,n}=\left\{\begin{array}{cc} r_{0} & \;\;n=1,\\ \sqrt{\frac{2n-2}{2N_{r}-3}}\;r_{\max}, & \;\;n=2,\ldots ,N_{r}, \end{array}\right.\;m=1,\ldots ,N_{a},$$

(2) $$\theta_{m,n}=\frac{\ln\;\left(\frac{r_{m,n}}{r_{0}}\right)}{\cot v}+2\pi \;\frac{m-1}{N_{a}},\;n=1,\ldots ,N_{r}\;\;m=1,\ldots ,N_{a},$$

respectively. Note that the angular coordinate follows a logarithmic spiral characterized by the growth angle *v*, and the term $2\pi \left(m-1\right)/N_{a}$ introduces a uniform angular offset between spiral arms, enforcing circular symmetry and avoiding redundant inter-microphone spacings. The outer radius has been limited to $r_{\max}\leq 0.2\;m$. This limit was considered to provide a maximum trace length respecting the recommendation by the manufacturer and preventing any data loss.

**Figure 5:**
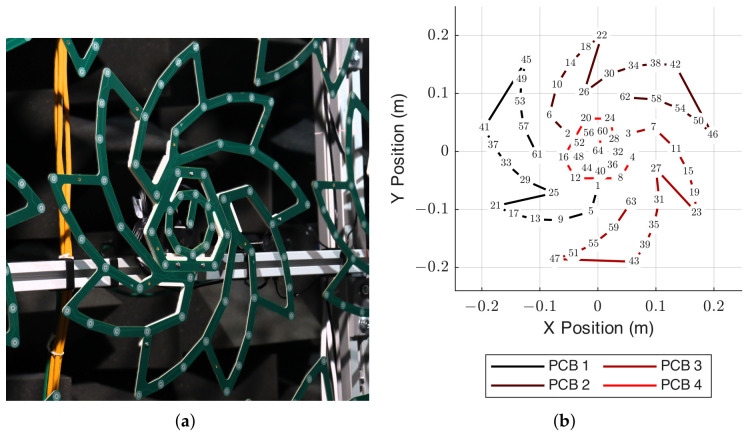
Sensor distribution. (**a**) The MxArray module consists of 64 MEMS microphones arranged in a spiral pattern along four PCBs. (**b**) The four PCBs forming the spiral determine the channel sorting for each McASP serializer.

Whilst the internal radius $r_{0}$ considers the minimum surface area occupied by each microphone, its associated components, and the sufficient space to maneuver the six pin connections around the microphone. Ergo, $r_{0}>\left(N_{a}\times 10\mathrm{mm}\right)/2\pi$. The BBB’s McASP peripheral can receive up to four multiplexed data lines simultaneously, forming a 64-element module. Thus, seven microphones ($N_{r}=7$) are distributed along nine arms ($N_{a}=9$) plus one microphone at the origin of the spiral, as depicted in Figure 5.

The PCB layout and the overall array geometry are based on both the length of the 16-channel data stream and the number of audio channels per module, which is limited by the number of multiplexed signals that the board’s SoC can handle simultaneously.

### 2.5. Interfacing Between the Control Logic and the Microphone Modules

**Figure 6:**
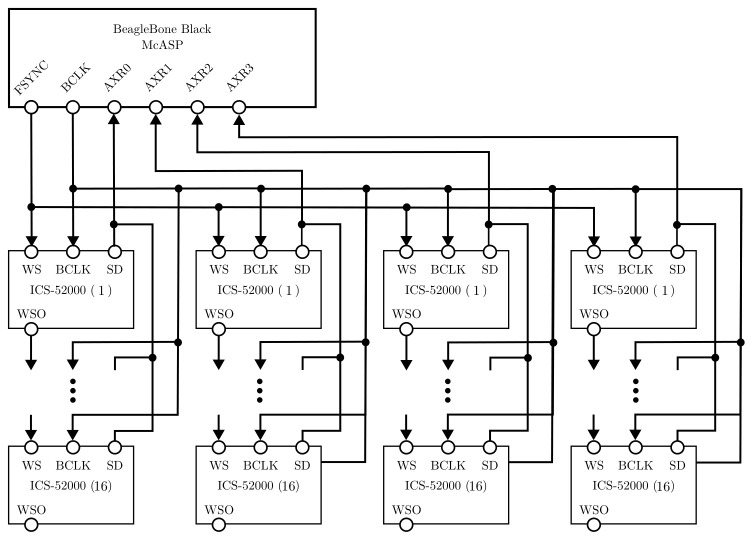
Sixty-four ICS-52000 microphones driving four separated TDM buses (SD) with sampling in sync. All sensors share a common serial clock (BCLK) from the processor’s audio peripheral. The McASP unit also provides a common word select (WS) signal to the first element of each daisy chain by outputting FSYNC.

As described in Section 2.2, each 64-microphone module is managed by a BBB single-board computer, which handles data acquisition, file transmission, and network synchronization. To simplify the connection and interfacing between the BBB and the four 16-microphone sub-modules that form a 64-microphone spiral array, a custom cape was designed to plug directly into the BBB’s expansion headers. The synchronization of the four SD buses is achieved following the manufacturer’s recommendations [9], as shown in Figure 6.

Each McASP interface is buffered using an OPA2810 operational amplifier, which provides sufficient drive strength and bandwidth to meet the system’s performance requirements. This component was chosen over a conventional clock buffer due to the need for bidirectional, separable signal driving: the FSYNC and BCLK lines are buffered from the BBB’s McASP ports toward the microphone pins, and the data line operates simultaneously in the opposite direction.

In addition, the cape functions as a power distribution node for the connected sub-modules: the 5 V rail that powers the BBB is split into a parallel branch and regulated down to 3.3 V using an LM1117-3.3 linear regulator capable of outputting around 800 mA, providing ample headroom for the four PCBs that comprise a 64-microphone module. Each 64-microphone module is estimated to draw approximately 0.5 W (i.e., about 150 mA at 3.3 V); the LM1117 therefore dissipates roughly 0.25 W from the 5 V to 3.3 V drop, which remains within safe thermal limits. The regulated 3.3 V supply is distributed using a star topology to minimize voltage drops and prevent ground loop formation, with each sub-board incorporating local decoupling capacitors to ensure stable operation. This distribution scheme provides sufficient current capacity and thermal margin to maintain a consistent state. Thus, each cape provides a complete interface solution, integrating signal buffering, power regulation, and connectivity between the BBB and the microphone sub-modules.

## 3. BBB Configuration

The default BBB pin configuration allows the microprocessor to offer various services, and although multichannel acquisition is possible, its synchronized clock signals and audio receivers are not fully enabled to receive TDM data streams. Therefore, we modified the BBB pin assignment, including the McASP TDM configuration and customized audio drivers and codecs. Because the McASP configuration is not dynamic, the kernel must load a compiled driver description file at boot to correctly initialize the pin assignments, clocks, and serial data paths. The easiest way to enable the McASP peripheral is by implementing a modular patch known as Device Tree Overlay (DTO).

### 3.1. Device Tree Overlay

The standard device tree describes the hardware layout of the BBB at boot, mapping the microprocessor pins and routing them to the BBB pin headers. In our specific case, the DTO file links the McASP peripheral with physical pins in the header, and additional General-Purpose Input/Output (GPIO) pin functions are also described (e.g., [18]). These pin assignments are also known as “pinmuxing” and can enable or disable hardware without rebuilding the kernel. To enable the McASP interface, the DTO file must describe the mode, direction, and PIN available to pinmux TDM synchronized clocks and data ports. The latter are also called serializers or audio transmit/receive (AXR).

As mentioned in Section 2.3, the quartz oscillator used for HDMI audio transmission can be internally rerouted and used as an external source clock. This clock signal feeds the McASP unit to generate BCLK and FSYNC. To link the clocks, they must be declared and divided by integers within the McASP node. Additionally, the TDM parameters must be declared with the number of bits per channel, the number of slots, and the number of serializers.

The Debian Linux distribution running on the BBB includes the open-source Advanced Linux Sound Architecture (ALSA) framework to manage audio hardware. To enable ALSA to correctly receive and capture multiplexed audio streams, the system must define the Digital Audio Interface (DAI) connections, including the links between the CPU, codecs, and audio paths. This configuration is specified through the simple-audio-card codec driver, which describes the expected connections between the McASP interface and the audio codec [19]. The MEMS microphone digitizes the acoustic signal using a sigma-delta (ΣΔ) modulator, effectively acting as an audio codec. As a result, the Linux kernel must be informed about the sampling frequency $f_{s}$, audio format, and number of channels provided by this so-called “Dummy Codec”, as detailed in this technical report [20].

### 3.2. Buffering Parameters

The bus bandwidth required for such a multiplexed acquisition must be carefully balanced with the system’s interrupt handling capabilities. To manage the throughput of such 64-channel acquisition, the parameters of the command-line utility, arecord, must be tuned to align the ALSA period and buffer sizes with Enhanced Direct Memory Access (EDMA) transfer thresholds [14]. The primary challenge is avoiding buffer overruns (Xruns), which occur when the CPU cannot consume audio data from the ALSA circular buffer as fast as the hardware fills the reserved memory with new data. These parameters must account for the 32-bit word length (4 bytes), the 8 kHz sampling rate, and the 64 multiplexed channels, resulting in a continuous data rate calculated as:

8000samples/s×4bytes×64channels=2,048,000bytes/s≈2MB/s.

The *Buffer Size* and *Period Size* are the two key parameters tailored to prevent system saturation. The Period Size defines the number of samples processed between hardware interrupts. This value must be large enough to reduce CPU overhead by increasing the time between processing cycles. Specifically, with a period of 1024 frames, the time available for the CPU to respond is:

$$\frac{1024\;\mathrm{frames}}{8000\;\mathrm{Hz}}=0.128\;s\;(128\;\mathrm{ms}).$$

While the buffer size parameter sets the total circular-buffer capacity, the system must also minimize computational operations that might slow down the CPU. This is achieved by using the memory mapping flag (—mmap), which allows the application to access the audio buffer directly. This bypasses expensive memory-copy operations between the kernel and user space, significantly reducing the processing load.

The choice of a buffer size that is four times the period size provides a critical safety margin. While the McASP hardware fills one period, the CPU has the duration of the remaining three periods to handle system tasks and commit data to RAM (/dev/shm). This multi-period configuration creates a “cushion”, providing a total safety window calculated as:

$$\mathrm{Total}\;\mathrm{Safety}\;\mathrm{Margin}=\left(\mathrm{Buffer}\;\mathrm{Size}-\mathrm{Period}\;\mathrm{Size}\right)\times \frac{1}{f_{s}}$$

$$\left(4096-1024\right)\times \frac{1}{8000}=0.384\;s\;(384\;\mathrm{ms}).$$

This 384 ms window was tested across several recordings, ensuring that, even during temporary CPU spikes, the acquisition process remained stable and the hardware continued to write to the circular buffer without overwriting unsaved data. Furthermore, to support these large requirements, modifications were made to the EDMA and DaVinci modules during the Kernel rebuilding to increase the internal buffer limits and ensure stable DMA (Direct Memory Access) transfers.

### 3.3. Real-Time Kernel and Driver Configuration

To achieve a synchronized modular acquisition, a custom Real-Time (RT) Kernel image was built. The build process involves cross-compiling a baseline kernel tree on a separate Linux workstation using the PREEMPT_RT patch set [21,22,23]. Beyond standard configuration, specific source code modifications were required within the *davinci-mcasp.c* driver to bypass the hardware limitations of the AM335x McASP module.

Specifically, the driver was modified to unblock the simultaneous reception of four serializers, enabling the 64-channel Time Division Multiplexed (TDM) stream. Furthermore, the Frame Synchronization (FSYNC) logic was reconfigured to meet the requirements of the TDM microphone; i.e., the FSYNC pulse is generated to be exactly one BCLK cycle wide and strictly synchronized with the BCLK falling edge, as illustrated in Figure 2. During kernel configuration (menuconfig), a Dummy Codec was integrated to provide a software abstraction for the hardware-level TDM stream. The resulting kernel image and DTO are deployed to the BBB, ensuring that the system bootloader (/boot/uEnv.txt) correctly initializes the RT environment and memory-coherent pools, reserving at least 8 MB of memory for DMA allocations.

## 4. MxArray Orchestration: PTP and MQTT

The array’s scalability relies on a combination of an Internet of Things (IoT) protocol and precise clock distribution over Ethernet. The embedded Linux system eases the connectivity among clients (modules) by using the network services.

Communication and control between modules is done via MQTT, better known as “mosquitto”. MQTT is an ISO-standard Machine-to-Machine protocol specifically engineered to be light and effective [24]. The message traffic is a broker-based publish–subscribe mechanism, creating a highly efficient, event-oriented architecture. The acquisition pipeline is illustrated in Figure 1, and it works as follows. Once all devices are set to a MQTT subnet, the broker unit (BBB 100) gets messages from the publisher (PC) and distributes them among clients (BBB 101 to 116) in the same channel or topic. The message published by the PC triggers a sequence of commands to activate the acquisition on each BBB. This process comprises: starting the recording with specific input parameters, assigning file names for each module, and triggering a synchronized time-reference pulse. This MQTT architecture allows for a controlled end-to-end command latency of 20–30 ms across the entire subnet, providing a robust method for orchestrating simultaneous actions without the overhead of individual TCP connections [24].

To ensure a shared temporal reference, the Precision Time Protocol (PTP) was implemented over the local network, designating a primary time server to distribute the reference clock. Since the BBB lacks a battery-backed Real-Time Clock (RTC), the Grandmaster (BBB 100) first synchronizes to a global reference via the Network Time Protocol (NTP) and then serves as the PTP Grandmaster. The slave modules listen on the eth0 interface, allowing the PTP Hardware Clock (PHC) to discipline each local oscillator. This ensures that while each module operates independently, they all share a common, nanosecond-accurate UTC timebase.

### Pulse Injection and Signal Alignment

While PTP ensures the system clocks are aligned, the execution of the arecord command remains stochastic. Initializing 64 channels involves complex tasks, such as connecting hardware to drivers, reserving DMA channels, and allocating RAM storage. This sequence introduces a variable startup jitter that could exceed about 100 ms, making simultaneous start-times impossible through software commands alone.

To overcome this, a pulse injection strategy was implemented using a high-priority, preemptive task. This secondary task is scheduled to trigger a 10 ms pulse via a dedicated GPIO (integrated as a pilot LED on the PCB) at a specific UTC timestamp in the future. By utilizing the SCHED_FIFO scheduler at maximum priority (99), the trigger task preempts non-critical kernel threads during the recording process, minimizing the scheduling jitter of the trigger execution. Consequently, regardless of when each modular arecord process was initiated, the recorded audio files across all modules contain a synchronized electrical marker. This allows for sample-accurate alignment during post-processing by detecting the leading edge of the injected pulse, effectively neglecting the latency related to the initial command execution.

## 5. Characterization Methodology

In the MxArray, the MEMS microphones digitize the signals and transmit them sequentially through buses designed to be as long as possible. The acquisition process can introduce delays that need to be characterized by evaluating each channel’s transfer function using broad-spectrum signal excitations, such as frequency chirps [25,26]. The procedure used for this characterization is described in the next subsection.

### 5.1. Exponential Sine Sweep

We implemented Farina’s measurement technique [27] to characterize complex sound systems. This methodology allows for computing Impulse Response (IR) by means of an Exponential Sine Sweep (ESS), represented as

(3) $$s\left(t\right)=sin\left[2\pi f_{1}L\left(e^{t/L}-1\right)\right]\;,$$

where $L=T/\ln\left(\frac{f_{2}}{f_{1}}\right)$, $f_{1}$ and $f_{2}$ are the lower and upper sweep frequency limits, respectively, and *T* is the total duration of the sweep in seconds.

Once s(t) is played through the electroacoustic chain, the captured ESS y(t) is subsequently deconvolved to obtain the IR as follows:

(4) h(t)=y(t)⊗f(t),

where ⊗ symbol represents the convolution operator, and f(t) is the inverse filter of the original sweep computed as

(5) $$f\left(t\right)=Ae^{t/L}sin\left[2\pi f_{1}L\left(e^{\frac{T-t}{L}}-1\right)\right].$$

**Figure 7:**
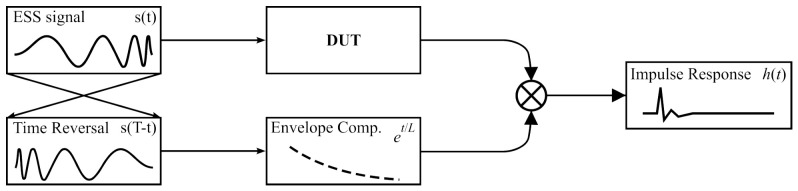
Block diagram illustrating the ESS technique for IR measurement. The excitation signal s(t) is recorded by the Device Under Test (DUT). The acquired output is subsequently convolved (⊗ operator) with an inverse filter, constructed by time-reversing the original sweep s(T−t) and applying an amplitude compensation envelope $e^{t/L}$, to recover the system’s IR h(t). Inside the boxes, the continuous lines represent temporal signals, and the dashed curve represents the modulator.

To distribute the energy concentrated in the low frequencies due to the exponential growth, the time-reversed ESS is convoluted with an envelope that ensures 6 dB per octave. This process, illustrated in Figure 7, yields the IR of the evaluated system.

### 5.2. Validation and Measurement Setup

A validation process was conducted to ensure the accuracy of the implemented ESS method. First, the method was benchmarked against a calibrated reference system. Second, the inherent latencies of the PCB MEMS-based hardware were quantified within an anechoic environment.

#### 5.2.1. Dirac vs. Non-Synchronized ESS Measurements

Since the TDM-based MEMS microphones are integrated directly into the PCB, they cannot be interfaced with standard acoustic measurement software such as Dirac^®^ Room Acoustics Software (version 7.3, Brüel & Kjær, Nærum, Denmark) via traditional analog audio inputs. Therefore, validation was achieved by performing a non-synchronized ESS measurements and comparing the IR obtained via Dirac system, which relies on a loopback hardware and synchronous recording to compensate for system latency, against the IR generated by our custom implementation.

**Figure 8:**
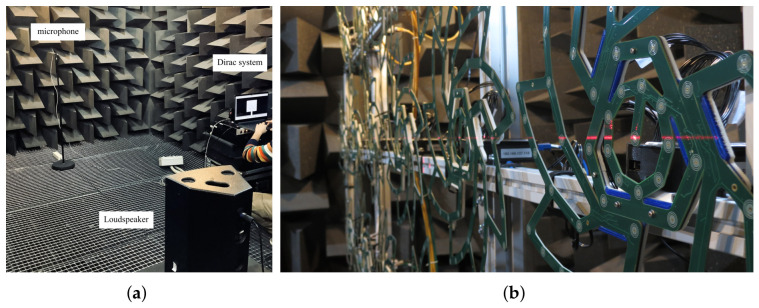
Equipment alignment for method validation, single module measurement, and delay estimation measurements. (**a**) Sweep acquisition using a precision microphone and a full-range speaker to generate the ESS with MATLAB^®^(version R2025a, The MathWorks, Inc., Natick, MA, USA) and with the Dirac^®^ commercial software (version 7.3, Brüel & Kjær, Nærum, Denmark). (**b**) MxArray alignment with the center of the speaker cone using a laser line level.

The validation setup was placed inside an anechoic chamber located in the Signal Theory and Communications Department at the Universidad Carlos III de Madrid (UC3M), utilizing a GRAS type 26AK (GRAS Sound & Vibration, Holte, Denmark) precision condenser microphone as the reference sensor. The excitation signal was reproduced by a full-range two-way passive speaker model TQ310 driven by a Crest Audio^®^ (CA6, Crest Audio Inc., Meridian, MS, USA) power amplifier. Signal conversion was managed by an RME Fireface UFX II audio interface at 44.1 kHz, selected for its high-fidelity ADC/DAC capabilities. The microphone was positioned at a fixed distance of 3 m from the sound source to ensure consistency in the acoustic far-field, as shown in Figure 8a. To protect the equipment from transient clipping during playback, a Hann window was applied to the beginning and end of the custom excitation sweep.

To identify and chop the portion signal with the ESS, a 1 kHz, pure-tone sync signal preceded the sweep. Applying cross-correlation of this sync tone allowed for the determination of the start and end points of the signal, effectively selecting the sweep for a direct IR comparison.

#### 5.2.2. Individual Module Latency Analysis

**Figure 9:**
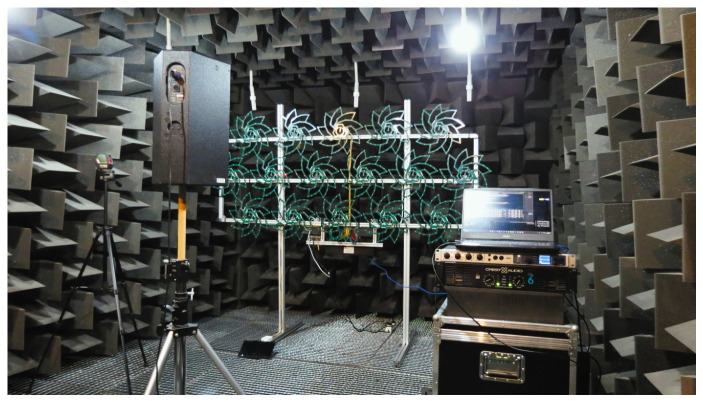
MxArray measurement setup for characterizing the module latency and network synchronization stability.

The first measurement campaign focused on characterizing the intrinsic delays within a single MxArray module, specifically to isolate delays from both the internal microphone operation and network latency. The MxArray module was aligned horizontally with the center of the speaker’s cone, maintaining the normal distance for all microphones of the module (see Figure 8b). The ESS signals were reproduced using the same electroacoustic chain as in the previous Section 5.2.1. Moreover, the 3 m distance was conserved, as displayed in Figure 9.

In this analysis, the signal captured by the $i^{\mathrm{th}}$ microphone belonging to the $m^{\mathrm{th}}$ module is denoted as $y_{i,m}\left(t\right)$, as shown in the next equation:

(6) $$y_{i,m}\left(t\right)=h_{i,m}\left(t-\tau_{\mu ,i,m}\right)⊗s\left(t-\tau_{\mathrm{geom},i,m}\right)+n\left(t\right),$$

where the term $h_{i,m}\left(t\right)$ represents the IR of the $i^{\mathrm{th}}$ channel, and s(t) stands for the ESS signal, delayed by the time-of-flight corresponding to the distance from the microphone position $\xi_{i,m}$ to the loudspeaker position $\xi_{LS}$. The last term, n(t), indicates the additive noise components which are usually attributed to acoustic background noise and/or the microphone’s inherent noise. The employment of a high excitation level combined with the high acoustic insulation of the facility ensures that the ESS signal is significantly elevated above the background level. Moreover, Farina’s method is recognized for providing inherent robustness against non-correlated noise. Consequently, the contribution of n(t) to the IR calculation is considered negligible relative to the ESS. In addition, the sound source emits such signal which undergoes several delays before reaching the digital register. For each microphone *i* belonging to the module *m*, the total temporal offset $\tau_{\mu ,i,m}$ encompasses the network and sensor latencies as an electronic latency due to the modular synchronization ($\tau_{\mathrm{NET},m}$) and the microphone’s transduction and ADC ($\tau_{\mathrm{ADC}}$), respectively. Consequently, the delay budget comprises the following components:

(7) $$\tau_{\mu ,i,m}=\tau_{\mathrm{NET},m}+\tau_{\mathrm{ADC}}+\epsilon_{i}.$$

Since we want to assess a single module, the network synchronization term is not taken into account ($\tau_{\mathrm{NET},m}=0$). Additionally, the comparison of the relative delays, which considers one microphone of the module as the reference, cancels out both common delays originated in the transduction and the ADC. Finally, the residual, $\epsilon_{i}$, represents small delay variations with respect to $\tau_{\mathrm{ADC}}$, revealing the delay deviation related to the aforementioned internal ASIC processes, which are unique for each microphone.

The propagation delay $\tau_{\mathrm{geom},i,m}$ is calculated using the Euclidean distance formed between the position of the sound source $\xi_{LS}$ and the microphone $\xi_{i,m}=\left(x_{i},y_{i},z_{i}\right)$ as:

(8) $$\tau_{\mathrm{geom},i,m}=\frac{\left|\right|\xi_{LS}-\xi_{i,m}\left|\right|}{c},$$

with the speed of sound c≈343m/s.

An alternative method for calculating the delay between channels is to extract the group delay from the impulse response. After extracting the IR $h_{i}\left(t\right)$ for each channel, the frequency domain representation $H_{i}\left(\omega \right)$ was obtained together with the phase response, $\phi_{i}\left(\omega \right)$, as follows:

(9) $$\phi_{i}\left(\omega \right)=\arg\left\{H_{i}\left(\omega \right)\right\}.$$

The time shift is determined by extracting the phase response, whose slope along the frequencies shows the delay group as

(10) $$\tau_{exp,i}=-\frac{1}{2\pi }\frac{d\phi_{i}\left(\omega \right)}{d\omega },$$

where $\phi_{i}\left(\omega \right)$ is the angle formed between the real and imaginary parts of the frequency response.

### 5.3. Network Synchronization Stability

**Figure 10:**
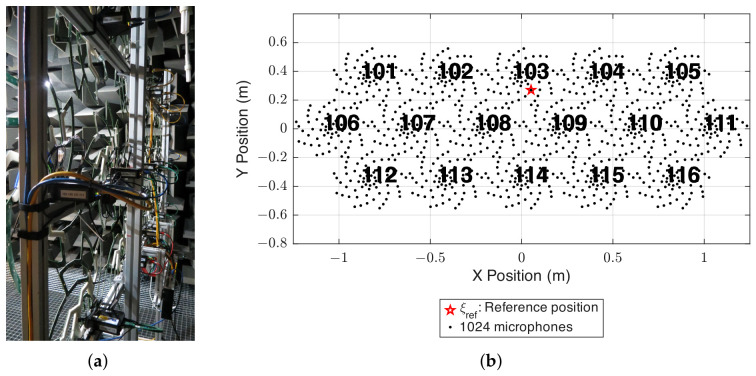
(**a**) Sixteen BBB clients are controlled by the BBB master, all connected to the main switch via Ethernet. The modules, including the BBB master, are fed current by an isolated power source at 5 V. (**b**) The honeycomb pattern allows stacking the modules. Geometrically, the nine arms of the spiral shape form a nonagon, which was considered as a circumscribed hexagon to fill the spans in the offset arrangement.

The complete 16-module array consisting of 1024 microphones is fixed on the aluminum frame shown in Figure 10a with their power and Ethernet connections in the rear side. The honeycomb module distribution, as displayed in Figure 10b, is aligned with the sound source, having as reference position the central microphone of module 3 since this position is the most aligned to the speaker. In this evaluation, the main focus is on network synchronization between modules, denoted as $\tau_{\mathrm{NET},m}$.

This delay is significantly influenced by both the ALSA command execution latency and the synchronization clock over the LAN, i.e., PHC. The first latency occurs between the moment when the command-line arecord is issued, and the first sample is transferred from the McASP peripheral into system memory via the EDMA controller. The time this process requires is estimated and compared with the theoretical arrival times of the same microphone element across all modules simultaneously. In order to quantify the jitter of the Linux kernel’s interrupt when initiating the recording command, a pulse injection method is implemented to align signals across all modules relative to a common external reference. The alignment of these audio files is subsequently performed as a post-processing task. This test uses the same speaker alignment and equipment as in the single module assessment, as depicted in Figure 8b. The horizontal axis of the microphone array was aligned to the center of the speaker’s cone, ensuring precise geometric delay estimation.

The synchronization error between the physical clocks of BBB clients and the Grandmaster remains within the nanosecond range within the LAN. This temporal offset is monitored by the PTP4l service, and it varies slightly at the BBB’s boot time. To quantify the clock synchronization among modules, the real-time offset of the 16 BBB clients was monitored with the Linux utility systemctl.

## 6. Results

### 6.1. IR Method Validation

**Figure 11:**
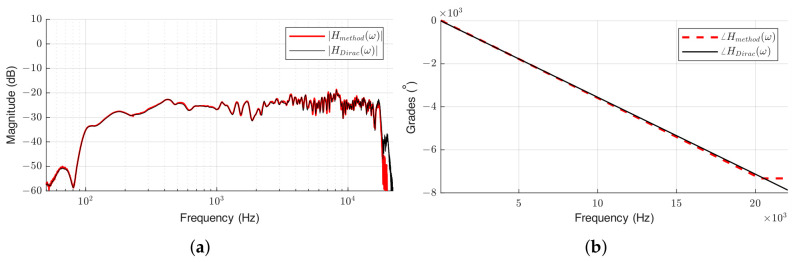
Validation of the custom ESS deconvolution method against the Dirac commercial software in an anechoic chamber. (**a**) Magnitude response comparison: custom method vs. Dirac reference using a GRAS microphone. (**b**) The unwrapped phase, represented in degrees, shows identical phase slopes, confirming the absence of algorithmic latency or artifacts. Hann windowing successfully prevents hardware clipping while maintaining phase linearity in the frequency range of interest.

In order to establish the ground truth, on which the rest of the results rely, the validation of the IR method demonstrates the spectral and temporal alignment of our custom method with that computed by the commercial software Dirac, using identical hardware. As shown in the frequency response comparison in Figure 11a, the magnitude |H(ω)| obtained from the custom method demonstrates high fidelity matching the reference curve across the effective bandwidth. A constant scaling offset was observed, likely due to Dirac’s internal normalization algorithms. The observed decay beyond 20 kHz of our custom method is a direct result of the excitation signal’s bandwidth limit ($f_{2}=20\;\mathrm{kHz}$), whereas the Dirac sweep extends to the Nyquist frequency ($f_{s}/2$). Most crucially for this study, the phase response analysis in Figure 11b shows a tight alignment comparing the unwrapped phase slopes between the two methods.

The unwrapped phase, ϕ(ω), shows a consistent linear trend, from which the sub-sample delay estimation is obtained. Additionally, the identical slopes confirm that the group delay is accurately captured without phase distortions. This experiment allows us to corroborate the correct operation of our implementation of Farina’s method.

### 6.2. Single Module Measurement

**Figure 12:**
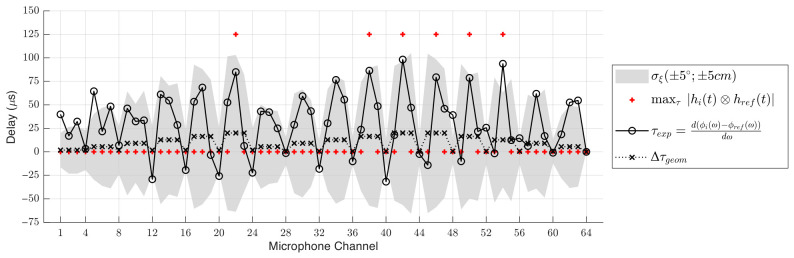
Comparison of estimated relative delays for a 64-channel module relative to the reference channel 64. The plot displays the residual delays after subtracting the theoretical time-of-flight for a source at 3 m. The experimental values for $\tau_{exp}$ are obtained via frequency-domain phase slope linear regression. Red crosses correspond to the time-domain cross-correlation peak-finding method ($\max_{\tau }\left|h_{i}\left(t\right)⊗h_{ref}\left(t\right)\right|$). The dotted line with black crosses tracks the nominal geometric delay $\Delta \tau_{\mathrm{geom}}$. The grey region $\sigma_{\xi }$ represents the geometric uncertainty bound derived from potential tilt ($\pm 5^{\circ }$), pan ($\pm 5^{\circ }$), and axial displacements (±5cm).

The experimental delay estimation for the single-module configuration is presented in Figure 12, considering microphone 64 as the reference. The most traditional method for estimating the relative delay between two signals is by calculating the cross-correlation (⊗ operator) and dividing the sample index of the maximum correlation by $f_{s}$. By comparing the results of the group delay, $\tau_{exp}$, against the traditional time-domain peak-finding method, it is evident that the frequency domain approach provides a more stable and precise estimation of sub-sample delays. With the module positioned at 3 m from the speaker, the relative theoretical time-of-flight, $\Delta \tau_{\mathrm{geom}}$, was calculated with the reference microphone 64, the $i^{\mathrm{th}}$ microphone, and source positions, yielding the following comparison between the theoretical and experimental results.

The position error $\sigma_{\xi }$ accounts for physical assembly tolerances that alter the distance between the source and each sensor. This region was calculated by applying rotation and translation matrices to account for potential tilt, panning, and axial displacements during the array assembly process. All measured residuals remain within the sub-sample regime (<125 μs), with minor deviations reflecting mechanical positioning and electric tolerances of each sensor.

### 6.3. Network Synchronization and Jitter Assessment

**Figure 13:**
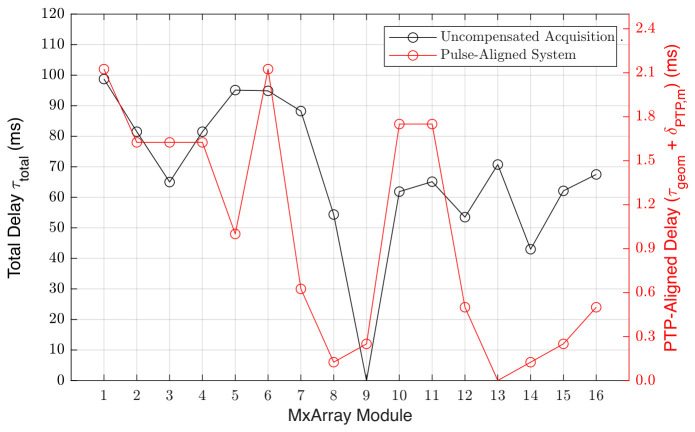
Comparative analysis of synchronization latency across the 16 MxArray modules. The black curve represents the raw arecord command execution latency (left axis), incorporating both geometric propagation and the stochastic delays inherent in standard OS command execution. The red curve (right axis) depicts the PTP-aligned delay using the pulse injection method, where the network jitter is reduced to a residual offset $\delta_{PTP,m}$ while preserving the deterministic geometric delay $\tau_{\mathrm{geom}}$. All values are normalized to the minimum observed delay.

The synchronization performance of the MxArray was evaluated by comparing the raw execution latency of the recording command against the aligned timing achieved via the pulse injection strategy (Section 4). Figure 13 illustrates the measurement across the 16 modular units. The raw arecord command execution (black line) exhibits a stochastic latency ranging up to approximately 100 ms relative to the start of the first module. This significant jitter is primarily attributed to the non-deterministic nature of the standard Linux scheduler and the varying initialization times required for multi-channel DMA buffer allocation. It is important to note that these values represent a single execution instance. Moreover, this latency changes stochastically with every run, making it impossible to align audio files without a consistent temporal reference.

To achieve the alignment of audio files between modules, the pulse injection method was utilized. By selecting the first sample captured from the pulse across the entire multiplexed data stream, the temporal drift was reduced. This approach accounts for the fact that the pulse may appear in any channel within the TDM stream, depending on the exact sample at which the GPIO was triggered. For this implementation, PCB number 2 was designated for pulse injection, resulting in the synchronization mark appearing on channels 2, 6, 10, up to channel 62.

The aligned results (red line) show a substantial reduction in jitter with residual delays normalized to the minimum observed value. The remaining offset of approximately 2.1 ms is not a synchronization error, but represents the embedded geometric delay inherent to the array’s physical configuration and position of microphone 62 in each module. This performance demonstrates that while the operating system introduces tens of milliseconds of software latency, the hardware-level trigger successfully disciplines the acquisition of a system that does not have a distributed clock.

### 6.4. PTP Network Synchronization

To evaluate the spatial fidelity of the synchronized MxArray, the geometric delay was estimated for all 1024 channels and compared against the theoretical geometric model. The Time-Domain Cross-Correlation method was applied to calculate the relative delay once all audio files were aligned with the pulse.

**Figure 14:**
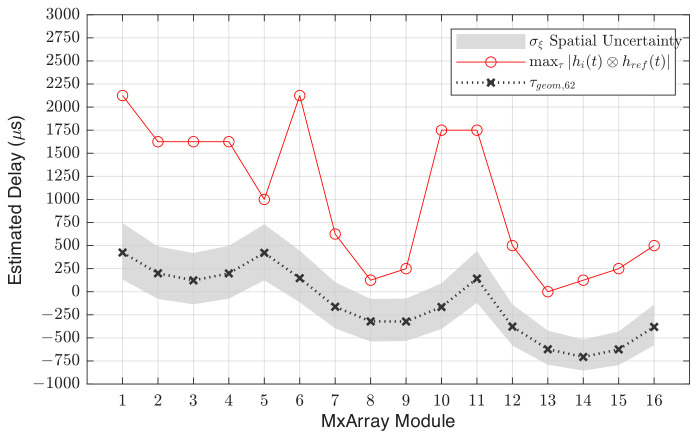
Comparative analysis of Time-of-Arrival estimation method for the 1024-channel array. The black dotted line with cross markers represents the theoretical geometric propagation delay. The red curve with circular markers depicts the relative delay estimated through the cross-correlation of the captured signals in the time domain. The shaded region $\sigma_{\xi }$ accounts for the spatial uncertainty in the physical positioning of the microphones.

As illustrated in Figure 14, the experimental results show a high degree of correlation with the geometric model ($\tau_{\mathrm{geom}}$). The reduced variance confirms that the PREEMPT_RT kernel and the pulse injection strategy successfully reduced the software-induced jitter.

On the other hand, the information extracted from the command-line utility systemctl details the measured clock offset of each BBB client with the BBB Grandmaster. Such a tool also provides the mean path delay due to the client-grandmaster length of cabling. Consequently, PTP controls the compensation applied to each BBB client. Table 1 shows the precision alignment with the Grandmaster clock (BBB 100) achieved by our system, where if the offset is negative (e.g., −32.0 ns for BBB 101), the client’s clock is "fast" relative to the Grandmaster BBB 100. Because these offsets fluctuate dynamically in real time due to network jitter and crystal thermal drift, Table 1 reports a representative operational snapshot that captures the worst-case scenario observed across multiple experimental runs.

The Mean Path Delay averaged approximately 13.3 μs. While this value represents the physical propagation time through the Ethernet cabling and network switches, it does not contribute to the synchronization error. Under the IEEE 1588 standard [28], the PTP protocol accurately estimates and compensates for this latency, ensuring that the system clocks are aligned regardless of cable length or network topology.

## 7. Discussion

The experimental validation confirms that the custom ESS deconvolution provides a reliable characterization of the MxArray, using Dirac software as the ground truth. Protection windowing (Hann) did not color the results. In fact, windowing the signal at the very beginning and end of the time-domain sweep has a negligible effect on the mid-band group delay, where the slope of the phase is linear. The experimental validation reported in Figure 11 clearly shows that the ESS estimation method can be considered accurate at least up to 20 kHz. Since final MxArray measurements are carried out at 8 kHz, the limit identified by the validation represents more than double the bandwidth of the final results, ensuring that the ESS method can be safely applied to the MEMS-based system.

The MxArray is theoretically capable of acquiring at up to 16 kHz. Nevertheless, a configuration of 8 kHz was selected for this characterization to ensure a robust operational overhead. Considering both the bus bandwidth required for bit-stream transfers and the 512 MB DDR3 RAM constraints, the system achieves stable acquisition without Xruns.

The propagation delay associated with signal transmission along the PCB traces is neglected. According to the microphone’s datasheet, a configuration equivalent to ours exhibits a propagation delay of 2 ns when the sampling rate is at 48 kHz for a common serial data trace as long as ours. This 2 ns delay is a fixed electronic property that remains constant at 8 kHz, becoming completely negligible due to the wider digital clock margins at this lower rate.

In Equation (7), we broke down the delay contributors, where the cumulative delay $\tau_{\mathrm{ADC}}$ could not be measured. However, the process accounts for the pressure wave transduction up to sending the binary stream in its respective slot, with a variation $\epsilon_{i}$ per microphone. The microphone’s ASIC uses a double-buffer to hold the data word until the WS signal has triggered to output onto the TDM bus. Indeed, the manufacturer states that a period of $2/f_{s}$ (250 μs at 8 kHz) is needed to synchronize and manage the TDM transmission [9]. Because this internal ASIC latency is identical for all channels, it cancels out for relative array measurements. However, it introduces an absolute, deterministic delay that must be compensated for when aligning the microphone data with external systems, such as cameras or other measurement systems.

The evaluation of the single module reveals that the majority of measured delays fall within a range attributed to mechanical positioning tolerances, represented by the gray region in Figure 12. However, specific units such as microphones 1, 3, 5, 48, and 54 exhibit deviations, with a maximum residual error of approximately $\epsilon_{i}$ = 25 μs after discounting the geometric positioning bias.

The acoustic impact of any transduction bias at a sampling rate of 8 kHz is calculated as

(11) $$\Delta \phi =360^{\circ }\times \epsilon_{i}\times f_{Nyquist}.$$

According to Van Trees [29], phase errors are negligible for beamforming applications if $\Delta \phi <5^{\circ }$ at $f_{Nyquist}$, i.e., approximately 3.5 μs. However, a phase error of up to $10^{\circ }$ is common in MEMS microphones due to the manufacturing tolerances inherent in low-cost devices. These sub-sample fluctuations do not preclude accurate beamforming. Since the observed delays are systematic and stationary, they can be corrected via calibration to ensure optimal array performance.

In the jitter reduction using the pulse injection, a systematic offset of approximately 0.2 ms is observed between the experimental data and the theoretical curve. This offset is attributed to the fixed hardware latency of the digital MEMS microphones and the TDM bus propagation. However, the consistency of this delay across all 16 modules validates the scalability of the synchronization architecture, ensuring that the entire dense array behaves as a single, phase-coherent aperture.

In the PTP synchronization metrics, the greatest clock offset shows a peak deviation of 438 ns (BBB 106), which corresponds to a phase shift of 0.63° according to Equation (11). As this maximum error is nearly an order of magnitude below the limit at the sampling rate we acquire, the synchronization error between modules is considered statistically irrelevant since this offset represents only 0.35% of the sampling period ($T_{s}=$ 125 μs).

## 8. Conclusions and Future Work

This work presented the development and validation of the MxArray, a 1024-channel microphone array based on a distributed network of 16 BeagleBone Black modules. The research demonstrates that massive data acquisition can be handled through an integrated embedded Linux approach. Thanks to its modular architecture, the cost per 64-channel module is remarkably low at approximately 300 euros, including all components, PCBs, capes, and assembly. This represents a substantial step toward affordability in high-density aeroacoustic sensing.

The core innovation of the MxArray lies in its inherent scalability. By combining TDM with McASP’s versatility over Ethernet-based protocols (PTP and MQTT), the array can be expanded by simply adding modules up to the capacity of the network switch. The PCB electronic design, combined with the non-redundant log-spiral sensor distribution, allows for massive spatial sampling that can be deployed in different module patterns.

The implementation of a synchronized pulse injection strategy, prioritized via the RT-scheduler, provided a hardware time marker across TDM data streams with sub-microsecond jitter. Experimental results confirmed that the phase-domain group delay remains stable across the entire aperture, ensuring the sub-sample phase relationships required for advanced spatial filtering. The modular nature of the MxArray, coupled with the efficiency of the MQTT-based control pipeline and the high-throughput McASP driver modifications, establishes a robust framework for high-resolution beamforming.

Future improvements of the MxArray will focus on further optimizing the network topology by replacing standard Ethernet switches with PTP-aware hardware. By designating the switch itself as the PTP Grandmaster, we can achieve even greater temporal stabilization across larger-scale, multi-switch deployments. Beyond hardware upgrades, we aim to transition from the current SCHED_FIFO trigger to a dedicated PRU-based firmware to eliminate residual kernel interrupt latencies. Furthermore, the system will incorporate a self-calibration framework based on supervised learning tools to map the relationship between impulse response features and sensor deviations. This machine learning approach aims to predict geometric and sensitivity corrections, thereby enhancing the overall spatial fidelity and beamforming performance of the massive array.

## Figures and Tables

**Table 1:** PTP synchronization metrics for the 16-node microphone array. Clock Offset $\delta_{PTP,m}$ is measured with the command-line ptp4l.service.

Node (BBB)	*δ*_*PTP*,*m*_ (ns)	Mean Path Delay (ns)
100 (GM)	0.0	0.0
101	−32.0	13,712.0
102	322.0	13,721.0
…	…	…
106	438.0	13,716.0
107	−120.0	13,727.0
108	−225.0	12,995.0
…	…	…
114	182.0	12,965.0
115	215.0	12,895.0
116	264.0	12,875.0

## Data Availability

The data are available from the authors upon reasonable request.

## Abbreviations

The following abbreviations are used in this manuscript:

MQTT	Message Queuing Telemetry Transport
McASP	Multichannel Audio Serial Port
TDM	Time-Division Multiplexing
MEMS	Micro-Electro-Mechanical System
PTP	Precision Time Protocol
PHC	PTP Hardware Clock
NTP	Network Time Protocol
ADC	Analog-to-Digital Converter
IC	Integrated Circuit
FPGA	Field-Programmable Gate Array
I2S	Inter-IC Sound
GPIO	General-Purpose Input/Output
PCB	Printed Circuit Board
BBB	BeagleBone Black
BCLK	Bit Clock
FSYNC	Frame Synchronization
$f_{s}$	Sampling Rate
WSO	Word Select Output
WS	Word Select
SD	Serial Data
SoC	System on Chip
DTO	Device Tree Overlay
ALSA	Advanced Linux Sound Architecture
DAI	Digital Audio Interface
DMA	Direct Memory Access
EDMA	Enhanced Direct Memory Access
IoT	Internet of Things
IR	Impulse Response
ESS	Exponential Sine Sweep
DUT	Device Under Test
LAN	Local Area Network
